# Selection and Validation of Reference Genes for Accurate RT-qPCR Data Normalization in *Coffea* spp. under a Climate Changes Context of Interacting Elevated [CO_2_] and Temperature

**DOI:** 10.3389/fpls.2017.00307

**Published:** 2017-03-07

**Authors:** Madlles Q. Martins, Ana S. Fortunato, Weverton P. Rodrigues, Fábio L. Partelli, Eliemar Campostrini, Fernando C. Lidon, Fábio M. DaMatta, José C. Ramalho, Ana I. Ribeiro-Barros

**Affiliations:** ^1^Plant-Environment Interactions and Biodiversity Lab (PlantStress&Biodiversity), Linking Landscape, Environment, Agriculture and Food, Departmento de Recursos Naturais, Ambiente e Território, Instituto Superior de Agronomia, Universidade de Lisboa (ULisboa)Oeiras, Portugal; ^2^Programa de Pós-Graduação em Genética e Melhoramento, Centro de Ciências Agrárias e Engenharias, Universidade Federal do Espírito SantoAlegre, Brazil; ^3^Setor Fisiologia Vegetal, Centro de Ciências e Tecnologias Agropecuárias, Universidade Estadual Norte Fluminense-Darcy RibeiroRio de Janeiro, Brazil; ^4^Departmento de Ciências Agrárias e Biológicas, Centro Universitário Norte do Espírito Santo, Universidade Federal Espírito SantoSão Mateus, Brazil; ^5^GeoBioTec, Departmento de Ciências da Terra, Faculdade de Ciências e Tecnologia, Universidade NOVA de LisboaMonte da Caparica, Portugal; ^6^Departmento de Biologia Vegetal, Universidade Federal ViçosaViçosa, Brazil

**Keywords:** climate changes, coffee, increased air [CO_2_], global warming, heat stress, normalization of transcriptomic studies, quantitative real-time PCR, reference genes

## Abstract

World coffee production has faced increasing challenges associated with ongoing climatic changes. Several studies, which have been almost exclusively based on temperature increase, have predicted extensive reductions (higher than half by 2,050) of actual coffee cropped areas. However, recent studies showed that elevated [CO_2_] can strongly mitigate the negative impacts of heat stress at the physiological and biochemical levels in coffee leaves. In addition, it has also been shown that coffee genotypes can successfully cope with temperatures above what has been traditionally accepted. Altogether, this information suggests that the real impact of climate changes on coffee growth and production could be significantly lower than previously estimated. Gene expression studies are an important tool to unravel crop acclimation ability, demanding the use of adequate reference genes. We have examined the transcript stability of 10 candidate reference genes to normalize RT-qPCR expression studies using a set of 24 cDNAs from leaves of three coffee genotypes (CL153, Icatu, and IPR108), grown under 380 or 700 μL CO_2_ L^−1^, and submitted to increasing temperatures from 25/20°C (day/night) to 42/34°C. Samples were analyzed according to genotype, [CO_2_], temperature, multiple stress interaction ([CO_2_], temperature) and total stress interaction (genotype, [CO_2_], and temperature). The transcript stability of each gene was assessed through a multiple analytical approach combining the Coeficient of Variation method and three algorithms (geNorm, BestKeeper, NormFinder). The transcript stability varied according to the type of stress for most genes, but the consensus ranking obtained with RefFinder, classified *MDH* as the gene with the highest mRNA stability to a global use, followed by *ACT* and *S15*, whereas α*-TUB* and *CYCL* showed the least stable mRNA contents. Using the coffee expression profiles of the gene encoding the large-subunit of ribulose-1,5-bisphosphate carboxylase/oxygenase (*RLS*), results from the *in silico* aggregation and experimental validation of the best number of reference genes showed that two reference genes are adequate to normalize RT-qPCR data. Altogether, this work highlights the importance of an adequate selection of reference genes for each single or combined experimental condition and constitutes the basis to accurately study molecular responses of *Coffea* spp. in a context of climate changes and global warming.

## Introduction

Impacts from recent climate-related extremes, such as heat and cold waves, droughts and strong rainfall events, reveal remarkable vulnerability of agricultural systems. Estimated future climate changes are expected to further amplify the existing climate-related risks and create new ones (IPCC, 2013). Within this context, it is important to assess crop ability to adapt their vital processes at a speed compatible with these environmental changes, allowing the selection and breeding of elite genotypes.

Coffee is one of the most important agricultural traded commodities, growing in more than 60 tropical countries. It is estimated that *ca*. 25 million farmers produce coffee in over 1 million ha, with a majority of smallholders whose livelihoods depend on this crop (Waller et al., 2007). In the last years, worldwide crop yields were above 8 million tons of green coffee beans (ICO, 2014a), generating an income of *ca*. US$ 173,000 million (ICO, 2014b), and involving approximately 100 million people considering the entire chain of value of coffee (Bunn et al., 2015).

Coffee production and quality are highly dependent on a regular sequence of climate events; temperature and water availability are considered the most important limiting environmental variables to this crop (DaMatta and Ramalho, 2006). Also, several modeling studies on the global impact of climate changes, mostly focused on increased air temperatures, have predicted reductions of suitable areas for Arabica coffee (up to *ca*. 50%) by 2,050 (Bunn et al., 2015; Magrach and Ghazoul, 2015), compromising the livelihoods of millions of small householders.

Under field conditions, the superimposition of environmental events is the most common situation, as is expected to be the case of elevated [CO_2_] and enhanced temperatures predicted along this century. In fact, depending on anthropogenic greenhouse gas emission scenarios, [CO_2_] might reach between 421 and 936 μL CO_2_ L^−1^ by 2,100, accompanied by a global warming up to between 2.6 and 4.8°C relative to 1986–2005 (IPCC, 2013, 2014). However, it was recently shown that increased [CO_2_] in the atmosphere strengthens the coffee plant by reinforcing its photosynthetic performance (Ramalho et al., 2013; Ghini et al., 2015; DaMatta et al., 2016). Furthermore, enhanced [CO_2_] has a crucial role in the mitigation of heat impact, and, therefore, in the resilience of coffee plant to supra-optimal temperatures, with positive repercussions ranging from mineral nutrition (Martins et al., 2014) to the triggering of defense mechanisms and altered gene expression (Martins et al., 2014; Rodrigues et al., 2016). Therefore, the catastrophic predictions on the future of the coffee crop, that are based almost exclusively on temperature drift (Rodrigues et al., 2016), should be reconsidered. In addition, results from Rodrigues et al. (2016) strongly suggest the need to study the single and superimposed effects of elevated [CO_2_] and supra-optimal temperatures at all plant levels, from morphology up to the molecular assessment.

The release of *Coffea* sp. expressed sequence tag (EST) databases (e.g., Poncet et al., 2006; Vieira et al., 2006; Mondego et al., 2011) and *Coffea canephora* genome (http://coffee-genome.org/) has greatly prompted the study of genes involved in important agronomic and stress tolerance traits, making marker-assisted selection a straight forward approach. Gene expression analysis is an important tool to elucidate the complex regulatory networks of the genetic, signaling, and metabolic mechanisms that underlie plant-environmental interactions (Mallona et al., 2010). Monitoring differential gene expression and validating high-throughput RNA sequencing (RNA-seq) data is ideally achieved through quantitative real-time PCR (RT-qPCR) analysis. However, accuracy and reliability of RT-qPCR relies on the normalization of gene expression data (Artico et al., 2010; Die et al., 2010). To avoid severe pitfalls in data analysis and interpretation, this implies the selection and systematic validation of suitable reference genes to be used as internal controls. In fact, expression stability should be validated for each particular plant tissue/organ, cell, and experimental conditions, particularly when involving environmental stressful conditions (Die et al., 2010; El Kelish et al., 2014; Imai et al., 2014; da Costa et al., 2015). In addition, the stability of reference gene transcripts is also species-dependent (Andersen et al., 2004; Gutierrez et al., 2008a,b), as in the case of coffee (Cruz et al., 2009; Goulao et al., 2012). A number of housekeeping genes (e.g., b-actin, elongation factor1a, 18S ribosomal RNA, and polyubiquitin) involved in general cell metabolism pathways are widely used to calibrate RT-qPCR studies in biological systems (Willems et al., 2008; Goulao et al., 2012; Imai et al., 2014; da Costa et al., 2015; Llanos et al., 2015). However, their expression levels may vary with the experimental conditions (Petriccione et al., 2015), and their use as internal reference genes should be taken with caution (Gutierrez et al., 2008a,b). For instance, *GAPDH* has been indicated as one of the most stable genes during single abiotic stresses (Tian et al., 2015), whereas it appeared to be the least stable gene during the plant development (Wang et al., 2016). In addition, the species-dependent stability of reference gene transcripts was observed, for example, in *TUB-A*, which is the most stable reference gene during celery development (Li et al., 2016) in contrast with what has been observed during the development of cherry (Ye et al., 2015).

In a context of advancing our knowledge regarding the plant responses to estimated climate changes and global warming, this work examined, for the first time, the transcript stability of candidate reference genes to accurately perform the normalization of RT-qPCR data from expression studies of *Coffea* spp. plants exposed to conditions mimicking predicted future environmental conditions. For that, single and combined impacts of elevated [CO_2_] and heat were considered.

## Materials and methods

### Plant material and growth conditions

Plant materials and experimental design was previously described in detail (Rodrigues et al., 2016). Briefly, three widely cropped coffee genotypes from the two main producing species were used: *Coffea arabica* L. cv. Icatu (an introgressed variety from *C. canephora* Pierre ex A. Froehner), *C. arabica* L. cv. IPR108, and *C. canephora* cv. Conilon Clone 153 (CL153). Potted plants of 1.5 years in age were transferred from a greenhouse (ambient [CO_2_]) into walk-in growth chambers (EHHF 10,000, ARALAB, Portugal) differing in air [CO_2_] supply: 380 μL CO_2_ L^−1^ (380-plants) or 700 μL CO_2_ L^−1^ (700-plants). Both groups of plants were then grown for 10 months in 28 L pots for *ca*. 10 months under controlled environmental conditions of temperature (25/20°C, day/night), RH (75%), irradiance (*ca*. 700–800 μmol m^−2^ s^−1^), photoperiod (12 h), without limitations of nutrients (Ramalho et al., 2013), water and space for root growth, the latter evaluated by visual examination at the end of the experiment, after removing the plants from their pots.

After that 10 months period, air temperature was gradually increased at a rate of 0.5°C day^−1^, from 25/20°C up to 42/34°C, with a 7 day stabilization at 31/25, 37/30 and 42/34°C to perform several evaluations (Martins et al., 2014, 2016; Rodrigues et al., 2016). Leaf material was collected from newly matured leaves, of both plagiotropic and orthotropic branches of the upper (illuminated) part of the plant, from five to eight plants per genotype and immediately frozen in liquid nitrogen and stored at −80°C until RNA extraction.

### Total RNA isolation and cDNA synthesis

Total RNA was isolated and quantified as described in Goulao et al. (2012) for each of the following 24 conditions: three genotypes (CL153, Icatu, IPR108), four temperature regimes (25/20°C, 31/25°C, 37/30°C, 42/34°C), and two [CO_2_] (380, 700 μL CO_2_ L^−1^), using the RNeasy Plant Mini Kit (Qiagen, Germany) according to the manufacturer's protocol. In order to avoid eventual genomic DNA contaminations, RNA samples were digested with Ambion® DNA-*free*™ DNase (Ambion, USA). RNA integrity was verified in 1.5% agarose—TAE gel electrophoresis containing GelRed Nucleic Acid Gel Stain (Biotium, USA). All RNA samples were individually analyzed for the possible presence of DNA contamination by standard PCR reactions (35 cycles) using primers designed for ubiquitin (UBQ) gene (Table 1), in the absence of cDNA synthesis. Total RNA concentration and purity were further verified through BioDrop Cuvette (BioDrop, UK) measurements to guarantee the same amount of starting material in subsequent cDNA synthesis. RNA concentrations ranged between 333 and 1,056 ng/μl, with OD 260:280 ratios always above 1.98.

**Table 1:** **Primer sequences and amplicon characteristics for each of the 10 candidate reference genes under evaluation**.

**Gene symbol**	**Primer sequence (5′–3′)**	**Gene name**	**Acession number**	**Amplicon size (bp)**
*UBQ2*	GATGATACTTGGCCCTGCAC CCTTCCCAGCTTGTCAATGT	Ubiquitin-conjugating enzyme E2	GR984245	142
*α-Tub*	GTGCATCTCCATCCACATTG GGTGTTGAAAGCGTCGTCT	Alpha-tubulin	GT009437	146
*S15*	CAATAGGGGCTTGAAGAGG TACGCTGCCAATCATCTCAG	40S ribosomal protein S15	GR987196	146
*DNAJ*	GGTGAAGCTGATGAAGCAC CCGGGCTGGGATTTAATAAG	Plant DNA J protein	GR986679	157
*MDH*	CCTGATGTCAACCACGCAACT GTGGTTATGAACTCTCCATTCAACC	Malate dehydrogenase	GW464198.1	100
*ACT*	AAGCTTGCCTATGTGGCTCTTG TCACTTGTCCATCTGGCAATTC	Actin	GT000704	100
*ELF-4A*	GGTTATGCGTGCTCTGGGTGAC ATGAACCCCACTGGAAAGAATG	Eukaryotic initiation factor 4α	GT71729	103
*CYCL*	AGCTCTACGCAGACACGACTCC GGTCGATCCTTTGAAGTGCAAG	Cyclophilin	GT007167	115
*EF1A*	CATTGTGGTCATTGGTCATGTC ACACGCTTGTCAATTCCTCCA	Elongation factor 1α	GR996930	87
*GAPDH*	AGGCTGTTGGGAAAGTTCTTC ACTGTTGGAACTCGGAATGC	Glyceraldehyde 3-phosphate dehydrogenase	DV692958	100

One microgram of DNA-free total RNA was used to synthesize first-strand cDNAs using oligo-(dT)_18_ primer and the SuperScript II first-strand synthesis system (Invitrogen, USA).

### Selection of reference gene sequences and primer design

A set of 10 candidate genes were selected, comprising several frequently used reference genes, based on previous reports in (a) coffee plants (Cruz et al., 2009; Goulao et al., 2012; Carvalho et al., 2013): actin (*ACT*), glyceraldehyde 3-phosphate dehydrogenase (*GAPDH*), eukaryotic initiation factor 4α (*Elf4A*), elongation factor 1α (*EF1A*), cyclophilin (*CYCL*), and malate dehydrogenase (*MDH*); and (b) other plants (Cseke et al., 2009; El Kelish et al., 2014; Watanabe et al., 2014; Yan et al., 2014): 40S ribosomal protein subunit 15A (_S15_), DNAJ-like (*DNAJ*), ubiquitin-conjugating enzyme E2(*UBQ2*), and a alpha tubulin (α*-TUB*). The corresponding gene sequences were retrieved from published literature and the NCBI databases (Cruz et al., 2009; Vidal et al., 2010; Mondego et al., 2011; Goulao et al., 2012; Carvalho et al., 2013). Primers were designed using Primer3 software (Rozen and Skaletsky, 2000). The length of the primers was set to be between 19 and 26 bp, with a GC content ranging from 45 to 60% and a melting temperature (*Tm*-value) between 62° and 65°C. Amplicon length ranges were set to be between 100 and 150 bp. The probability of formation of hairpin structures and primer dimerization was subsequently checked using the Oligo Calculator (version. 3.26) algorithm (Kibbe, 2007). Primer sequences are given in Table 1.

### Quantitative real-time polymerase chain reaction conditions

RT-qPCR reactions were performed in 96-well plates using iQ™ SYBR® Green Supermix (BioRad, USA). Reactions were prepared in volumes of 25 μl containing 150 ng of cDNA and 3 μM each primer, in 1 × iQ™ SYBR® Green Supermix. Reactions were run on the iQ™ 5 Real-Time Detection System (BioRad, USA) using the following parameters: hot start activation of the TaqDNA polymerase at 95°C for 10 min, followed by 40 cycles of denaturation 95°C for 15 s; annealing at 60°C for 30 s; elongation at 72°C for 30 s; and plate read. To verify the specificity of each amplification, and the absence of primer dimers, dissociation curves were obtained for each amplicon at the end of the PCR run, by continuous fluorescence measurement from 55° to 95°C, with sequential steps of 0.5°C for 15 s. Three technical replicates were used for each biological replicate and the mean Ct was used for data analyses. The full sample set was always included in each technical replicate to exclude any artifacts consequential of between-run variations. No signals were detected in non-template controls run in parallel for each primer set. Two negative controls were included for each primer pair, in which cDNA was replaced by water or total RNA.

### Calculation of PCR efficiencies

Five-fold serial dilutions (1:1–1:625) of pooled cDNAs, which included equal-molar quantities of all samples independently for each genotype, were quantified in triplicates to generate standard curves for each primer pair. Based on the slopes of the standard curves, the PCR efficiency of each gene, for each genotype, was determined from the respective logarithm of the cDNA dilution, plotted against the mean threshold cycle (*Ct*) values. The reaction efficiency was calculated using the equation: E (%) = (10−(1/slope)−1) × 100 (da Costa et al., 2015), where E is the efficiency, in percentage, and slope is the gradient of the best-fit line in the linear regression.

### Selection of reference genes

The global transcript variability of each candidate reference gene was analyzed through statistical parameters using Box Plot. The variation of gene expression was calculated by means of coefficient of variation (CV), where *CV* = (standard deviation mean) × 100. These analyzes were performed using the statistical program R Core Team (2016). In order to select the best reference genes for the experimental conditions, expression stability was analyzed using geNorm (Vandesompele et al., 2002), NormFinder (Andersen et al., 2004) and BestKeeper (Pfaffl et al., 2004). The final rank of reference genes was determined with the RefFinder program (Xie et al., 2012), a web-based user-friendly comprehensive tool that integrates geNorm, Normfinder, BestKeeper, and the comparative Ct method.

### Determination of the minimum number of reference genes for data normalization

The pairwise variations (*V*-values) were calculated using the final ranking determined by RefFinder, through pairwise variation (V_n_/V_n+1_) of two consecutively serial log_2_-transformed normalization factor (NF) ratios, based on stepwise addition of the subsequent more stable reference gene (NF_n_ relative to NF_n+1_ reference genes; Vandesompele et al., 2002). The SD of the array generated by the log_2_-transformed NF ratios was calculated for each NF combination (V_n_/V_n+1_). V_n_/V_n+1_ were then plotted, representing changes in expression stability of NF. The optimal number of reference genes to exact standards was determined by calculating values as a pair of variation (Vn/Vn + 1) between two normalization factors consecutively classified (NC) after the gradual addition of more subsequent stable reference gene (NFN and NFN + 1), as proposed by Vandesompele et al. (2002), included in GeNorm package. A cut off value <0.15 was adopted (da Costa et al., 2015; Niu et al., 2015; Petriccione et al., 2015; Yang et al., 2015).

### Validation of reference gene analysis

The evaluation of the transcript levels from the RLS gene, encoding the ribulose 1,5-bisphosphate carboxylase/oxygenase (RuBisCO) large subunit, along the imposed temperatures (25/20°C, 31/25°C, 37/30°C, 42/34°C), and under both growth [CO_2_] (380, 700 μL CO_2_ L^−1^) was analyzed by RT-qPCR, using the most stable vs. the least stable genes (using one, two or three). The RT-qPCR amplification conditions were the same as described above. The relative expression data and statistical analysis were performed using the REST 2,009 software developed by Pfaffl (Technical University Munich) and Qiagen (Germany). Rest analysis takes into account PCR efficiencies and normalization gene, using the test hypothesis P (H1), i.e., the probability of difference between target and control groups be by chance. P (H1) performs at least 2,000 times of random reallocations of target samples and controls between the groups. Statistical differences were considered significant when *P* < 0.001 (^*^^*^^*^), *P* < 0.01 (^**^), and *P* < 0.05 (^*^).

## Results and discussion

**Figure 1:**
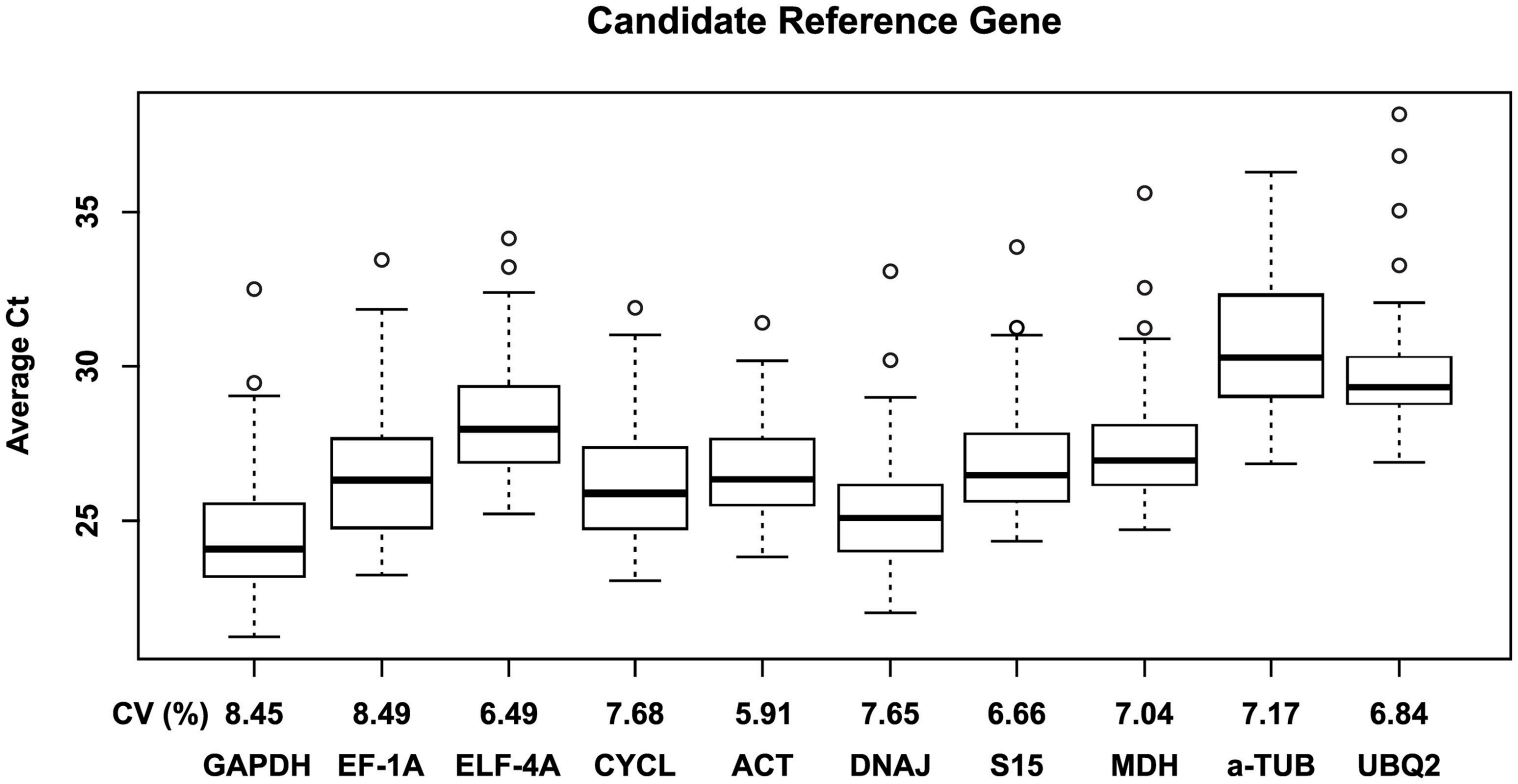
**Box plot representing the expression profiling variations of each candidate reference gene**. The primer pairs of each gene were used to examine all samples (*n* = 24; Table 1). *Boxes* indicate the 25th and 75th percentiles; *lines across the box* represent the median *Ct*-values; *whisker caps* represent the minimum and maximum values; and *spots* represent the outliers.

A total of 10 candidate genes (CG) to obtain an accurate validation of RT-qPCR data were assessed considering the individual comparison groups, i.e., genotype, [CO_2_] and temperature, multiple stress interaction ([CO_2_] and temperature) and total stress interaction (genotype, [CO_2_] and temperature). To determine the specificity of the primer pairs used in this study, melting curve analysis was performed after 40 cycles of amplification. The single peak obtained confirmed the specificity of the amplicon. No signal was detected in the negative controls of all tested primers. The standard curve method using a pool of all cDNA samples was performed to calculate RT-qPCR efficiency (E) and the correlation coefficient (R^2^) of each primer pair (Table 2). The raw quantification cycle (*Ct*) values ranged from 21.2 to 38.2 across all of the tested samples, with their minimal and maximal means falling between 24.1 ± 1.8 and 34.1 ± 2.1 (Figure 1). The results showed that *GAPDH* and α*-TUB* were the most and least abundantly expressed genes with mean Ct of 24.4 and 30.7, respectively. *EF-1A* showed the most variable levels of expression. Collectively, these results indicate that the transcript levels of the several candidate genes (CG) varied across different experimental samples confirming the need to screen the most appropriate reference genes for each experimental condition to accurately normalize RT-qPCR gene expression data. This result is common in plants given that most reference genes are involved in functions other than basal cell metabolism (Goulao et al., 2012; da Costa et al., 2015).

**Table 2:** **Efficiency values and correlation coefficient for each candidate reference genes under evaluation**.

**Gene**	**RT-qPCR efficiency% (*****R***^**2**^**)**		
	**CL153**	**Icatu**	**IPR108**
*GAPDH*	93.3(0.99)	95.7(0.98)	93.9(0.98)
*EF-1A*	96.3(0.99)	82.7(0.99)	97.3(0.96)
*ELF-4A*	90.8(0.99)	90.9(0.98)	93.3(0.96)
*CYCL*	87.1(0.96)	80.3(0.98)	80.3(0.98)
*ACT*	91.4(0.93)	98.7(0.93)	97.9(0.91)
*DNAJ*	82.5(0.99)	86.4(0.99)	97.7(0.98)
*S15*	99.9(0.96)	98.8(0.98)	90.0(0.95)
*MDH*	80.3(0.99)	92.0(0.96)	92.1(0.97)
*a-TUB*	99.3(0.96)	93.4(0.98)	94.0(0.99)
*UBQ2*	98.1(0.97)	91.2(0.93)	92.2(0.94)

According to the Coefficient of Variation (CV) method, the most stable genes were *ACT* (for genotype and total stress, *CV* = 5.91 in both cases), *UBQ2* (for temperature, *CV* = 5.50 and [CO_2_], *CV* = 5.71), and *S15* (for multiple stress, *CV* = 5.58; Table 3). *EF-1A* was ranked as the least stable gene for genotype (*CV* = 8.49), temperature (*CV* = 8.34) and overall stress (*CV* = 8.49), ranking in the 6th place for [CO_2_] and multiple stress. *DNAJ* and *TUB* were the less stable genes for [CO_2_] (*CV* = 9.68) and multiple stress treatments (7.95). Because the CV method alone is not sufficiently accurate to estimate the stability of the mRNA levels (Goulao et al., 2012), a complementary analysis was performed based on three algorithms, geNorm, NormFinder, and BestKeeper. According to the geNorm algorithm, the average expression stability (*M*-value) of all CG was below the default limit of 1.5 for genotype, temperature, [CO_2_] and their respective interactions, therefore indicating a considerable high stability level (Table 4). *GAPDH* was consistently classified as the most stable gene for the five comparison groups while *CYCL* was the least stable gene for all groups with the exception of genotype, where *MDH* was the least sable gene. On the other hand, the ranking obtained with NormFinder was clearly dependent on the comparison group: *MDH* was the most stable for genotype, *UBQ2* for temperature, *GAPDH* for [CO_2_] and *ELFA* for multiple and total stress groups. Such differences are due probably to different stability measures, i.e., while geNorm calculates gene expression stability based on the average pairwise expression ratio, NormFinder estimates the overall expression variation to provide individual stability values (Kozera and Rapacz, 2013). Nevertheless, similarly to geNorm, NormFinder also ranked *CYCL* as the least stable gene for all groups, with the exception for genotype (where α-TUB was the least stable CG).

**Table 3:** **Ranking of candidate reference genes according to its coefficient of variation (CV%), considering the variables genotype, temperature, [CO_2_] and their interaction**.

**Rank**	**Genotypes**		**Temperature**		**[CO**_**2**_**]**		**Multiple stress**		**Total stress**	
	**Gene**	**CV**	**Gene**	**CV**	**Gene**	**CV**	**Gene**	**CV**	**Gene**	**CV**
1	*ACT*	5.91	*UBQ2*	5.50	*UBQ2*	5.71	*S15*	5.58	*ACT*	5.91
2	*ELF-4A*	6.49	*S15*	5.82	*ACT*	5.73	*ELF-4A*	5.72	*ELF-4A*	6.49
3	*S15*	6.66	*ACT*	5.87	*ELF-4A*	5.76	*ACT*	6.19	*S15*	6.66
4	*UBQ2*	6.84	*MDH*	6.15	*MDH*	6.64	*MDH*	6.25	*UBQ2*	6.84
5	*MDH*	7.04	*ELF-4A*	6.38	*S15*	6.65	*DNAJ*	6.69	*MDH*	7.04
6	*α-TUB*	7.17	*DNAJ*	6.54	*EF-1A*	6.86	*EF-1A*	7.15	*a-TUB*	7.17
7	*DNAJ*	7.65	*CYCL*	6.75	*α-TUB*	6.96	*CYCL*	7.33	*DNAJ*	7.65
8	*CYCL*	7.68	*GAPHD*	6.93	*CYCL*	7.52	*UBQ2*	7.44	*CYCL*	7.68
9	*GAPHD*	8.45	*α-TUB*	7.22	*GAPHD*	8.94	*GAPHD*	7.74	*GAPHD*	8.45
10	*EF-1A*	8.49	*EF-1A*	8.34	*DNAJ*	9.68	*α-TUB*	7.95	*EF-1A*	8.49

**Table 4:** **Stability of candidate reference genes according to GeNorm, NormFinder and BestKeeper, considering the variables genotype, temperature, [CO_2_], and their interaction**.

**Group**	**Ranking**	**geNorm**		**NormFinder**		**BestKeeper**	
		**Gene**	**Stability**	**Gene**	**Stability**	**Gene**	**R**
Genotype	1	*GAPDH*	0.24	*MDH*	0.005	*ACT*	0.97^***^
	2	*ACT*	0.26	*DNAJ*	0.009	*MDH*	0.97^***^
	3	*EF-1A*	0.27	*UBQ2*	0.010	*ELF-4A*	0.96^***^
	4	*ELF-4A*	0.28	*S15*	0.011	*S15*	0.95^***^
	5	*UBQ2*	0.29	*ELF-4A*	0.013	*DNAJ*	0.93^***^
	6	*α-TUB*	0.32	*ACT*	0.016	*UBQ2*	0.93^***^
	7	*S15*	0.33	*EF-1A*	0.020	*GAPDH*	0.93^***^
	8	*CYCL*	0.42	*CYCL*	0.026	*CYCL*	0.85^***^
	9	*DNAJ*	0.53	*GAPDH*	0.027	*α-TUB*	0.82^***^
	10	*MDH*	0.62	*α-TUB*	0.030	*EF-1A*	0.81^**^
Temperature	1	*GAPDH*	0.14	*UBQ2*	0.041	*MDH*	0.98^***^
	2	*UBQ2*	0.14	*ELF-4A*	0.057	*DNAJ*	0.97^***^
	3	*ELF-4A*	0.16	*GAPDH*	0.060	*UBQ2*	0.96^***^
	4	*ACT*	0.16	*DNAJ*	0.071	*ACT*	0.96^***^
	5	*S15*	0.17	*EF-1A*	0.076	*S15*	0.95^***^
	6	*α-TUB*	0.17	*α-TUB*	0.082	*ELF-4A*	0.94^***^
	7	*DNAJ*	0.18	*ACT*	0.083	*EF-1A*	0.92^***^
	8	*EF-1A*	0.18	*MDH*	0.084	*GAPDH*	0.91^***^
	9	*MDH*	0.19	*S15*	0.086	*CYCL*	0.86^***^
	10	*CYCL*	0.37	*CYCL*	0.246	*α-TUB*	0.84^***^
[CO_2_]	1	*GAPDH*	0.15	*GAPDH*	0.019	*CAS15*	1.00^***^
	2	*UBQ2*	0.16	*ELF-4A*	0.030	*ACT*	0.99^***^
	3	*ELF-4A*	0.17	*UBQ2*	0.036	*MDH*	0.99^***^
	4	*S15*	0.18	*S15*	0.070	*ELF-4A*	0.98^***^
	5	*α-TUB*	0.18	*EF-1A*	0.076	*UBQ2*	0.98^***^
	6	*ACT*	0.19	*α-TUB*	0.077	*GAPDH*	0.97^***^
	7	*EF-1A*	0.19	*ACT*	0.082	*DNAJ*	0.94^**^
	8	*MDH*	0.23	*MDH*	0.105	*α-TUB*	0.91^*^
	9	*DNAJ*	0.31	*DNAJ*	0.180	*EF-1A*	0.91^*^
	10	*CYCL*	0.53	*CYCL*	0.357	*CYCL*	0.47
Multiple stress	1	*GAPDH*	0.16	*ELF-4A*	0.028	*MDH*	0.97^***^
	2	*ELF-4A*	0.16	*UBQ2*	0.053	*DNAJ*	0.96^***^
	3	*α-TUB*	0.17	*α-TUB*	0.060	*S15*	0.96^***^
	4	*ACT*	0.18	*GAPDH*	0.062	*ELF -4A*	0.96^***^
	5	*UBQ2*	0.18	*DNAJ*	0.074	*ACT*	0.95^***^
	6	*S15*	0.18	*ACT*	0.077	*EF-1A*	0.90^***^
	7	*DNAJ*	0.20	*S15*	0.082	*UBQ2*	0.90^***^
	8	*MDH*	0.21	*MDH*	0.094	*GAPDH*	0.88^***^
	9	*EF-1A*	0.27	*EF-1A*	0.164	*CYCL*	0.83^***^
	10	*CYCL*	0.43	*CYCL*	0.288	*α-TUB*	0.50^*^
Total stress	1	*GAPDH*	0.16	*ELF-4A*	0.040	*MDH*	0.97^***^
	2	*UBQ2*	0.18	*UBQ2*	0.044	*S15*	0.96^***^
	3	*ELF-4A*	0.18	*GAPDH*	0.055	*ELF-4A*	0.95^***^
	4	*S15*	0.19	*S15*	0.085	*ACT*	0.95^***^
	5	*ACT*	0.19	*ACT*	0.095	*DNAJ*	0.94^***^
	6	*α-TUB*	0.22	*DNAJ*	0.105	*UBQ2*	0.93^***^
	7	*DNAJ*	0.23	*MDH*	0.111	*GAPDH*	0.92^***^
	8	*MDH*	0.23	*α-TUB*	0.112	*CYCL*	0.82^***^
	9	*EF-1A*	0.24	*EF-1A*	0.123	*EF-1A*	0.79^***^
	10	*CYCL*	0.44	*CYCL*	0.295	*α-TUB*	0.73^***^

For sake of simplicity, only significant differences were indicated (

*** P < 0.001;

** P < 0.01;

* *P < 0.05) related to the BestKeeper test*.

In order to consolidate the data obtained with geNorm and NormFinder, we have included in the analysis the BestKeeper algorithm, which calculates the expression stabilities of the CG in accordance with *SD* and *CV* values. The calculated results classified *ACT* as the most stable gene for the genotype (together with *MDH*) and *S15* for the [CO_2_] group. For temperature, multiple stress and overall stress groups the most stable gene was *MDH*. α*-TUB, EF-1A*, and *CYCL* were ranked as the least stable genes, always below the 6th place and usually in the three last positions. Despite the differences between the ranking obtained with the CV method and the three algorithms, which are related to the individual classification criteria, *CYCL* and α*-TUB* were consistently ranked in the bottom five positions.

The consensus ranking obtained with RefFinder (Table 5), classified *MDH* as the most stable gene for all comparison groups, therefore constituting a good choice for the studies of *Coffea* spp. under future estimated conditions of increased [CO_2_] and temperature. Furthermore, *ACT* and *S15* were always ranked in the top five positions, while α*-TUB* and *CYCL* were amongst the least stable genes, as previously found in previous tests (Tables 3, 4). *MDH* and *ACT* have been previously described as being amongst the reference genes with higher transcript stability for RT-qPCR studies in *Coffea* spp. subjected to N starvation, salt and heat stresses (Carvalho et al., 2013) and to cold and drought stresses (Goulao et al., 2012), respectively. However, there was no consensus for the majority of candidate reference genes, i.e., the expression stability strongly varied according to the environmental stressful condition, again highlighting the importance of a proper and accurate selection of reference genes for each single experimental condition.

**Table 5:** **Overall ranking of the most stable genes within each treatment made through RefFinder program, considering the variables genotype, temperature, [CO_2_] and their interaction**.

**Genotypes**		**Temperature**		**[CO**_**2**_**]**		**Multiple stress**		**Total stress**	
*MDH*	1.68	*MDH*	1.86	*MDH*	1.68	*MDH*	1.41	*MDH*	1.41
*S15*	1.86	*UBQ2*	1.86	*S15*	1.78	*ELF-4A*	2.21	*ACT*	1.57
*ACT*	2.99	*DNAJ*	2.11	*UBQ2*	2.71	*DNAJ*	2.63	*S15*	2.78
*DNAJ*	3.34	*ACT*	3.46	*ACT*	2.83	*S15*	2.78	*DNAJ*	3.94
*ELF-4A*	3.83	*S15*	3.98	*ELF-4A*	4.40	*ACT*	4.40	*ELF-4A*	4.95
*UBQE2*	5.05	*ELF-4A*	6.24	*GAPDH*	6.82	*GAPDH*	6.64	*UBQ2*	5.05
*GAPDH*	7.00	*GAPDH*	6.74	*EF-1A*	7.00	*UBQ2*	6.74	*GAPDH*	7.00
*CYCL*	8.00	*EF-1A*	8.24	*a-TUB*	8.00	*CYCL*	8.00	*CYCL*	8.00
*EF-1A*	9.00	*CYCL*	8.74	*CYCL*	8.80	*EF-1A*	9.15	*a-TUB*	9.24
*a-TUB*	10.00	*a-TUB*	10.00	*DNAJ*	9.00	*a-TUB*	9.24	*EF-1A*	9.74

**Figure 2:**
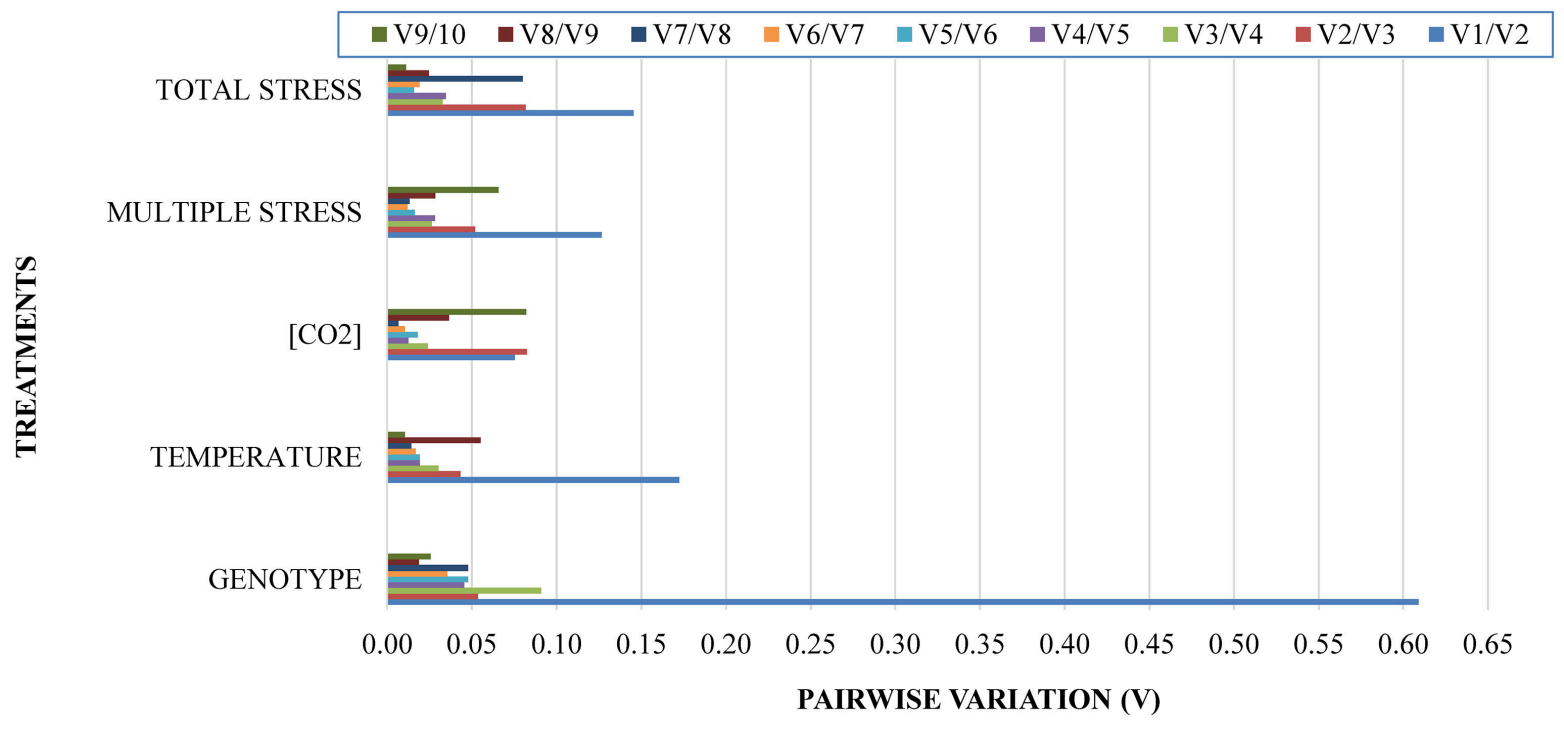
**Pairwise variation analysis to determine the optimal number of reference genes for RT-qPCR data normalization**.

For all comparison groups at V2/V3 the Pairwise variation (*V*) value was already below 0.15 (Figure 2), indicating that the use of two genes guarantee a strong accuracy for the normalization of RT-qPCR data. It is noteworthy to mention that for all treatments the pairwise variation values were <0.10 (except for the use of just one reference gene), indicating low expression instability of the selected genes tested for these environmental conditions.

In order to validate the consensus ranking, we have examined the relative expression of *RLS*, encoding the large-subunit of RuBisCO. The choice of this enzyme was related to its central role in the C-assimilation pathway, considering that improved crop photosynthetic efficiency under high temperature might be achieved through the replacement of a number of candidate amino acid substitutions to improve RuBisCO performance (Orr et al., 2016). Moreover, the *RLS* gene was selected based on the fact that, according to structural-functional analyses, most of the important amino acid residues necessary for catalysis are present in the large subunits (Morita et al., 2014). The consensus ranking established by RefFinder identified *MDH, UBQ2*, and *DNAJ* as the best stable genes to temperature, whereas *a-TUB, CYCL* and *EF-1A* where the least stable ones. Given that the increase in atmospheric temperature has proven to be the most disturbing variable to metabolism performance, mineral nutrition and acclimation limitation under predicted future environmental conditions (Martins et al., 2014, 2016; Rodrigues et al., 2016), with particular strong negative implications to RuBisCO catalytic ability (Rodrigues et al., 2016), we decided to use the most/least stable genes to temperature for *RLS* validation.

The expression profiles were normalized with the two and three most and least stable genes (Tables 6, 7), being observed that the expression patterns of *RLS* varied with the comparison group. Noticeably, due to the high *SD* of *Ct* values in many cases variations in *RLS* expression along the stress imposition were not reported as statistically significant. The patterns of *RLS* expression were nearly the same in RT-qPCR reactions calibrated with two or three genes. Independently of the stability of *RLS* mRNA, the increase in temperature generally resulted in up-regulation of *RLS* expression at standard [CO_2_] of 380 μL CO_2_ L^−1^ in all genotypes, except in IPR108 at 31/25° and 37/30°C where *RLS* expression was nearly the same as the control. For the 700 μL CO_2_ L^−1^ comparison group, the use of the most stable reference genes produced different results from those obtained with the least stable genes at 25/20° and 31/25°C. Thus, when reactions were normalized with the most stable genes, a consistent decrease in the steady-state amounts of *RLS* mRNA (which is the net result of transcription and degradation) was observed at these temperatures in all genotypes, while the use of the least stable genes indicated a positive regulation of *RLS* expression. For 37/30°C and 42/34°C, normalization of RT-qPCR with the most or the least stable genes produced similar up-regulated expression patterns of *RLS*. Notably, such an up-regulation occurred when RuBisCO activity begin to decrease (37/30°C) or was severely depressed (42/34°C) (Rodrigues et al., 2016). Such apparent discrepancy was also noted in chloroplastic ascorbate peroxidase (APX). APX activity was the most reduced (to about 10% of the control) at 42/34°C among the studied enzymes, contrasting to the largest increase in *APX* transcripts amongst all studied genes (Martins et al., 2016). Such large increases in gene transcripts may be related to an attempt of the plants to compensate the severe decline of enzyme activity, and further highlights the need of integrated experiments that include gene expression and enzyme activity studies.

**Table 6:** **Analysis of *RLS* expression by RT-qPCR, calibrated with the two most (*MDH* and *UBQ2*) and the two least (*a-TUB* and *CYCL*) genes for the temperature group/treatment**.

**Reference genes**			**MDH and UBQ2**	**a-TUB and CYCL**
**Genotype**	**Temperature**	**[CO_2_]**	**RLS expression**	**RLS expression**
CL 153	25/20°C	380	1.00	1.00
		700	0.361	1.768
	31/25°C	380	1.353	5.665
		700	0.504	3.559
	37/30°C	380	1.581	12.556
		700	1.467	12.86
	42/34°C	380	4.565	25.922
		700	8.889	25.114
Icatu	25/20°C	380	1.00	1.00
		700	0.781	1.553
	31/25°C	380	1.900^**^	3.604^*^
		700	1.505	2.546^*^
	37/30°C	380	3.423^*^	6.957^*^
		700	2.776^*^	5.742^*^
	42/34°C	380	5.220^*^	19.923^*^
		700	7.097^*^	32.153^*^
IPR 108	25/20°C	380	1.00	1.00
		700	0.249^*^	0.603
	31/25°C	380	0.778	1.345
		700	0.801^*^	1.720^*^
	37/30°C	380	0.844	1.851^***^
		700	1.070	2.172
	42/34°C	380	4.940^*^	21.732^**^
		700	1.399	4.01

For sake of simplicity, only significant differences were indicated (

*** P < 0.001;

** P < 0.01;

* *P < 0.05)*.

**Table 7:** **Analysis of *RLS* expression by RT-qPCR, calibrated with the three most (*MDH, UBQ2* and *DNAJ*) and the three least (*a-TUB, CYCL* and *EF-1A*) genes for the temperature group/treatment**.

**Reference genes**			**MDH, UBQ2, and DNAJ**	**α-TUB, CYCL and EF-1A**
**Genotype**	**Temperature**	**[CO_2_]**	**RLS expression**	**RLS expression**
CL 153	25/20°C	380	1.00	1.00
		700	0.410	1,347
	31/25°C	380	1,424	2,780
		700	0.522	1,747
	37/30°C	380	1,730	5,701
		700	1,435	6,357
	42/34°C	380	4,214	10,853
		700	6,527	10,782
Icatu	25/20°C	380	1.00	1.00
		700	0.744	1, 449
	31/25°C	380	1.608^*^	2.807^**^
		700	1,215	2.170^*^
	37/30°C	380	2.726^*^	5.250^*^
		700	2.219^*^	3.955^*^
	42/34°C	380	4.087^*^	9.637^*^
		700	5.389^*^	12.979^*^
IPR 108	25/20°C	380	1.00	1.00
		700	0.249^*^	1, 059
	31/25°C	380	0.697^*^	1.307^*^
		700	1,123	2.865^*^
	37/30°C	380	0.759	1,495
		700	1, 093	3,453
	42/34°C	380	4.950^*^	11.012^*^
		700	1,759	4,645

For sake of simplicity, only significant differences were indicated (

^***^P < 0.001;

** P < 0.01;

* *P < 0.05)*.

Given the facts described above, the reference genes used in this study were quite stable under the applied experimental conditions, as pairwise variation values were <0.10. The results obtained in the groups 700-25/20°C and 700-31/25°C call up for the importance to select the best reference genes. Altogether, despite the low instability of *a-TUB, CYCL*, and *EF-1A*, the use of reference genes showing a high stability of their mRNA amount is the best option for the correct interpretation of data from transcriptional studies (Roy et al., 2011; Kozera and Rapacz, 2013). Also importantly, this corroborates with previous studies in *Coffea* sp., which indicate that the selection of reference genes for an accurate RT-qPCR normalization should be performed accordingly to each for specific condition (Cruz et al., 2009; Goulao et al., 2012; Carvalho et al., 2013; Yuyama et al., 2016).

## Conclusions

In summary, our study reinforces the importance of the *Minimum Information for Publication of Quantitative Real-Time PCR Experiments* (MIQE) guidelines (Bustin et al., 2009) to normalize RT-qPCR data. The consensus ranking obtained with RefFinder showed that the transcript stability of the evaluated reference candidate genes changed according to the conditions/interactions, although *MDH* was the best one in all treatments, therefore, constituting a good common basis for RT-qPCR data normalization. However, the Pairwise variation analysis showed the need to use two reference genes, with the second most stable gene changing according to the genotypes and imposed environmental conditions: *S15* for genotype and [CO_2_], *UBQ2* for temperature, *EJF-4A* for multiple stress, and *ACT* for multiple stress. Using the expression profiles of coffee *RLS*, results from the *in silico* aggregation and experimental validation confirmed that the use of two reference genes is an adequate procedure to normalize RT-qPCR data.

## Author contributions

According to their competences, all authors contributed transversally to the several stages of the work, including its design, data acquisition, analysis and interpretation, critically review of the manuscript, and approval of the submitted version. Furthermore, they agree to be accountable for all aspects of the work in ensuring that questions related to the accuracy or integrity of any part of the work were appropriately investigated and resolved.

## Funding

This work was supported by by national funds from Fundação para a Ciência e a Tecnologia through the projects PTDC/AGR-PRO/3386/2012, the research units UID/AGR/04129/2013 (LEAF) and UID/GEO/04035/2013 (GeoBioTec), as well through the grant SFRH/BPD/47563/2008 (AF) co-financed through the POPH program subsidized by the European Social Fund. Brazilian funding from CAPES (grants PDSE: 000427/2014-04, WR; 0343/2014-05, MM), CNPq and Fapemig (fellowships to FD, FP, and EC) are also greatly acknowledged.

### Conflict of interest statement

The authors declare that the research was conducted in the absence of any commercial or financial relationships that could be construed as a potential conflict of interest.

## Abbreviations

RT-qPCR: quantitative real-time polymerase chain reaction.
